# Antithrombotic Use Patterns in COVID-19 Patients from Spain: A Real-World Data Study

**DOI:** 10.3390/jcm13082403

**Published:** 2024-04-20

**Authors:** Karen Lizzette Ramirez-Cervantes, Salvador Campillo-Morales, Patricia García-Poza, Manuel Quintana-Díaz, Consuelo Huerta-Álvarez

**Affiliations:** 1 Spanish Association Against Cancer, Division of Cancer Prevention, C/Teniente Coronel Noreña 30, 28045 Madrid, Spain; 2 Patient Blood Management Research Group, Hospital La Paz Institute for Health Research, 28040 Madrid, Spain; salvador.campillo@idipaz.es; 3 Division of Pharmacoepidemiology and Pharmacovigilance, Spanish Agency for Medicines and Medical Devices (AEMPS), C/Campezo n° 1, Edificio 8, 28022 Madrid, Spain; pgarcia@aemps.es; 4 Intensive Care Unit, Patient Blood Management Research Group, Research Institute of La Paz University Hospital, La Paz University Hospital, 28040 Madrid, Spain; 5 Department of Public Health and Maternity Childcare, Faculty of Medicine, Complutense University of Madrid, Pl. de Ramón y Cajal, s/n, 28040 Madrid, Spain; mahuer05@ucm.es

**Keywords:** COVID-19, thrombosis, prescription patterns, use rates

## Abstract

Antithrombotics have been widely used to treat and prevent COVID-19-related thrombosis; however, studies on their use at population levels are limited. We aimed to describe antithrombotic use patterns during the pandemic in Spanish primary care and hospital-admitted patients with COVID-19. **Methods:** A real-world data study was performed. Data were obtained from BIFAP’s electronic health records. We investigated the antithrombotic prescriptions made within ±14 days after diagnosis between March 2020 and February 2022, divided their use into prior and new/naive groups, and reported their post-discharge use. **Results:** We included 882,540 individuals (53.4% women), of whom 78,499 were hospitalized. The median age was 44.7 (IQR 39–59). Antithrombotics were prescribed in 37,183 (4.6%) primary care subjects and 42,041 (53.6%) hospital-admitted patients, of whom 7505 (20.2%) and 20,300 (48.3%), respectively, were naive users. Prior users were older and had more comorbidities than new users. Enoxaparin was the most prescribed antithrombotic in hospitals, with higher prescription rates in new than prior users (2348.2, IQR 2390–3123.1 vs. 1378, IQR 1162–1751.6 prescriptions per 10,000 cases, *p* = 0.002). In primary care, acetylsalicylic acid was the most used antithrombotic, with higher use rates in prior than in naïve users. Post-discharge use occurred in 6686 (15.9%) subjects (median use = 10 days, IQR 9-30). **Conclusions:** Our study identified a consensus on prescribing antithrombotics in COVID-19 patients, but with low use rates in hospitals.

## 1. Introduction

The hypercoagulable state of COVID-19 generates a profoundly prothrombotic environment, usually accompanied by abnormal coagulation parameters, which is associated with higher rates of venous thromboembolism (VTE), worse disease progression, and significant morbidity and mortality [1,2,3,4].

Anticoagulants have emerged as a potential treatment strategy to prevent and treat thrombosis in patients with COVID-19 [5,6]. For instance, thromboprophylaxis with low-molecular-weight heparin (LMWH) or unfractionated heparin (UFH) is indispensable in hospital-admitted patients. However, they are not recommended for non-hospitalized individuals [7,8]. In addition, anticoagulants used on the wards are not generally continued post-discharge to prevent thromboembolisms, except in cases of high risk of thrombosis or if different indications exist [9].

Benefits of antiplatelet therapy for preventing thromboembolic events in COVID-19 patients have yet to be demonstrated; thus, present-day recommendations are against their use during hospitalization [10]. However, subjects with underlying conditions receiving anticoagulant or antiplatelet therapies before COVID-19 should continue using them unless bleeding or other contraindications are present [10].

Monitoring how antithrombotics were prescribed during the pandemic is essential; however, large population-based studies are limited. In 2020, a global survey conducted on 515 physicians from 41 countries aimed to explore the management of COVID-19-associated coagulopathy found that 78% of professionals recommended thromboprophylaxis in all hospitalized patients [11,12]. In 2021, a retrospective study conducted in Spain reported that 85% of critically ill patients received a prescription for anticoagulant therapy with enoxaparin as the most prescribed medication [12]. Similarly, a recent investigation reported that anticoagulants accounted for most of the prescriptions performed in suspected and confirmed COVID-19 patients admitted to an ICU [13].

To our knowledge, population-level investigations on trends in antithrombotic prescription patterns during the pandemic have yet to be conducted in Spain; therefore, our study aimed to perform a real-world data analysis of antithrombotic use in the Spanish population with COVID-19.

## 2. Materials and Methods

### 2.1. Study Design and Ethics

We performed a real-world data study with a retrospective, observational, and transversal design. We investigated the use of antithrombotics in primary care and hospitals in patients with COVID-19 between 3 January 2020 and 28 February 2022.

### 2.2. Source of Data

Data were obtained from BIFAP, a longitudinal population-based database fully financed by the Spanish Agency for Medicines and Medical Devices (AEMPS is its acronym in Spanish) that has collaborated with the Autonomous Regions and has the support of the main scientific societies involved. BIFAP integrates health data from the electronic medical records (EMR) of more than 22 million anonymized medical histories from 15.373 primary health care practitioners and pediatricians (PCP) [14]. In terms of drug utilization, prescriptions issued by the PCPs are automatically recorded. In addition, from 2011 onwards, e-prescription has progressively been implemented in primary care centers; therefore, dispensation is also available. Prescriptions from specialists and those used during hospitalizations may not be fully captured; however, during the pandemic, specific data on hospital drug prescriptions were also gathered from Aragón, Asturias, Castilla y Leon, and Murcia, four of ten autonomous communities (ACs) currently integrating data on BIFAP. Thus, the present study captured data from these ACs. Patients’ personal information was anonymous. Details on BIFAP have been previously described [14].

### 2.3. Study Population

The inclusion criteria were as follows: patients had to be adults (≥18 years old) and have at least one year (≥365 days) of enrolment in BIFAP when they met the criteria. Those with a diagnosis of SARS-CoV-2 recorded in the database between 1 March 2020 and 28 February 2022 were identified (index date). Our target population included subjects with COVID-19 treated in primary care facilities and hospitals, including individuals admitted to intensive care units (UCIs).

Subjects with a date of hospital/ICU admission and/or a date of hospital/ICU discharge were considered hospitalized individuals. In contrast, individuals without hospital/ICU admission or discharge were considered patients from primary care. The length of hospitalization was not investigated.

We studied subjects’ baseline characteristics (present at the time of COVID-19 diagnosis) such as gender, age (stratified as 18–25, 25–40, 40–55, 55–70, and ≥70 years old), and comorbidities (obesity, cardiovascular diseases, coronary artery disease, cancer, coagulation defects, brain disorders of vascular etiology, acute liver disease, chronic kidney disease [CKD], chronic obstructive pulmonary disease [COPD], asthma, multiple sclerosis, Parkinson disease, dementia, Alzheimer’s disease, and inflammatory bowel disease [IBD]). We also searched for other medicines prescribed six months before the index date.

### 2.4. Outcomes

Our primary outcome was the antithrombotic use patterns in COVID-19 patients treated in primary care facilities and hospitals during the first six waves of the pandemic (2020–2022). Secondary outcomes included post-discharge antithrombotic use and duration, explored in patients receiving antithrombotics during hospitalization.

### 2.5. Antithrombotic Use

Using the datasets of EMRs of BIFAP, we searched for antithrombotic prescriptions based on their anatomical therapeutic chemical classification (ATC) codes assigned by the Collaborating Center for Drug Statistics Methodology of the World Health Organization (Table S1).

We accounted for the number of antithrombotic prescriptions made at any time after the index date (date of COVID-19 diagnosis). Still, for this analysis, we only considered the antithrombotic prescriptions made within the first ±14 days after diagnosis. We categorized users as new/naive users if they received antithrombotic agents exclusively within ±14 days from the index date, and as prior users if, besides this period, they received them within −15 to −365 days from diagnosis.

A 40-day follow-up period was established to investigate post-discharge antithrombotic use. If an antithrombotic was prescribed within this period, another 40-day follow-up period was examined. Post-discharge follow-up ended if no antithrombotic prescriptions were found within 40 days.

### 2.6. Statistical Analysis

A descriptive analysis was performed. We first accounted for the number of positive COVID-19 cases diagnosed between 1 March 2020 and 28 February 2022 and stratified them by periods of 14 days. Then, we divided the whole study period according to the COVID-19 waves in Spain. Information on waves was gathered from the National Epidemiological Surveillance Network (RENAVE) of the National Epidemiology Center; however, the beginning and end of each wave were adapted to the 14-day periods of our study. The dates did not change substantially but were set as follows: first wave, February 2020–22 May 2020, adapted to March 2020–31 May 2020; second wave, 6 July 2020–18 November 2020, adapted to 1 July 2020–30 November 2020; third wave, December 2020–16 February 2021, adapted to 1 December 2020–15 February 2021; fourth wave, 2 April 2021–17 May 2021, adapted to 1 April 2021–15 May 2021; fifth wave, 1 July 2021–29 September 2021, adapted to 1 July 2021–30 September 2021; and sixth wave, 13 November 2021–28 March 2022, adapted to 1 November 2021–28 February 2022.

Use rates were calculated as the average prescriptions per 10,000 COVID-19 cases. To do so, we divided the number of prescriptions for each drug in question (numerator) by the number of positive cases diagnosed in every 14-day period (denominator). The 14-day use rate and the average use rate were determined. The latter was determined by summing all the 14-day use rates and dividing them by the total 14-day periods.

Regarding post-discharge use, we calculated the median prescription durations and determined their 25–75 interquartile ranges (IQR). We also calculated the types of antithrombotics prescribed and the number of patients who received them.

We described the use rates of antithrombotics stratified by age, sex, and comorbidities. They were calculated for the whole study period and the subgroups of prior and new users from hospitals and primary care.

Categorical variables are represented as absolute or relative frequencies, whereas numeric variables are reported as means and standard deviations (SDs) or medians and interquartile ranges (IQRs), according to their distribution. We used the Shapiro–Wilk test to determine the normality of quantitative variables.

## 3. Results

### 3.1. Study Population

**Figure 1:**
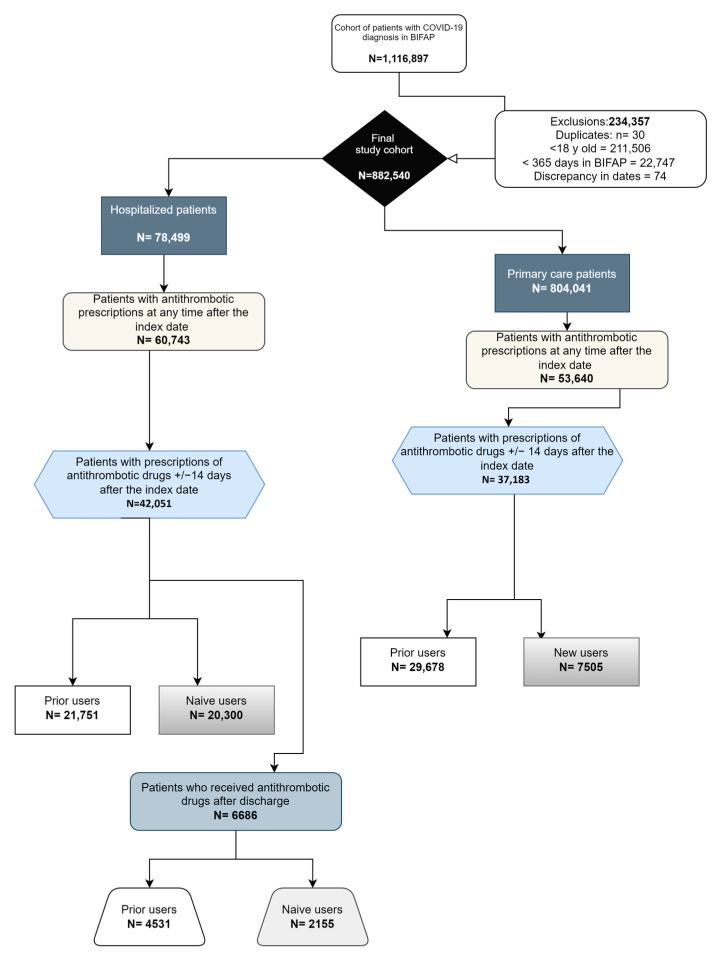
Description of the study population based on their antithrombotic use.

We identified 1,116,897 patients with COVID-19 in the BIFAP records; we eliminated 211,506 due to being ≤18 years old, 22,747 due to having enrolment periods <365 days in BIPAF, 74 due to having date inconsistencies, and 30 due to duplicate cases. Hence, our final study cohort comprised 882,540 individuals. Of them, 804,041 (91.1%) were only treated in primary care centers and 78,499 (8.9%) in hospitals (Figure 1).

In 2021, the total population was 1,326,261 in Aragon, 1,011,792 in Asturias, 2,383,139 in Castilla y Leon, and 1,518,486 in Murcia. Thus, the population of the present study is considered representative of these regions.

**Figure 2:**
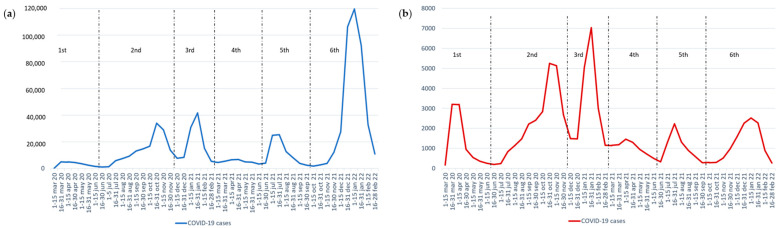
(**a**) COVID-19 cases in primary care during the first six waves of the pandemic, *n* = 804,041. (**b**) COVID-19 hospital cases during the first six waves of the pandemic, *n* = 78,499.

The distribution of COVID-19 cases during the six waves of the pandemic in our study period (1 March 2020 to 28 February 2022) can be visualized in Figure 2. As observed, the highest peak of cases in primary care occurred during the sixth wave. In contrast, there were more hospitalizations during the third wave, coinciding with the omicron variant.

Women accounted for most of the study population (53.4%) (Table 1). Females were also the majority among the primary care patients (52.6%). On the contrary, males accounted for most of the hospitalized individuals (55.5%) (Table S2). The gender distribution was similar in non-users (53.9%), users from primary care (52.6%), and post-discharge users (55.5%); however, among new users from primary care, the frequency of women was notably high (67.7%). Conversely, men accounted for most of the hospital-admitted population (55.5%) (Table 1).

The median age was 45 (IQR 33–59), and most of the population were 25–55 years old (56.6%); however, age ranges differed among subgroups. For instance, most prior users from primary care (58.9%) and hospitals (70.7%), as well as post-discharge users (63.6%), were aged ≥70 years. In contrast, new users were younger, as 55.6% of the primary care population were between 25 and 55 years old, and 57.5% were between 40 and 70 on the wards (Table 1).

### 3.2. Comorbidities and Other Prescriptions at Baseline

Comorbidities were more frequent among prior than naïve users from primary care and hospitals. The most common were cardiovascular diseases (82.3% vs. 27.5%, and 79.7% vs. 41.4%, *p* ≤ 0.05, respectively), obesity (22.8% vs. 12.9%, *p* ≤ 0.05 and 24.6% vs. 17.6%, *p* = 0.05, respectively), stroke (18.7% vs. 0.8% and 16.4% vs. 1.1%, *p* ≤ 0.05, respectively), and cancer history (14.1% vs. 5.5% and 19.8% vs. 9.1%, *p* ≤ 0.05, respectively) (Table 1).

Comparing prior and new users from primary care and hospitals, the use of other medicines before COVID-19 was represented mainly by agents acting on the renin–angiotensin system (51.5% vs. 14% and 50.2% vs. 25.4%, respectively), lipid modifying agents (56.5% vs. 12.4% and 46.9% vs. 20.6%, respectively), antidiabetics (29% vs. 5.9% and 30.8% vs. 12.1%, respectively), diuretics (27.1% vs. 5% and 42.3% and 10.5%, respectively), and drugs for obstructive airway diseases (15.2% vs. 8.8% and 28.9% vs. 15.9%, respectively) (Table S3).

Six months before the index date, the use of antibacterials was almost two times more frequent in hospital users (52.2%) than in their counterparts from primary care (27.8%). In addition, the use of antidepressants was more than doubled in prior users compared to new users from both primary care (24.4% vs. 10.3%) and hospitals (29.7% vs. 13.3%). Similarly, the previous use of psycholeptics (including antipsychotics, anxiolytics, hypnotics, and sedatives) was more frequent among prior than naïve users, considering non-hospitalized (40.7% vs. 18.9%) and hospitalized individuals (53.2% and 28.2%) (Table S3).

### 3.3. Antithrombotic Use Patterns

From the total study cohort (*n* = 882,540), 803,306 were considered non-users (without antithrombotic prescriptions within ±14 days from the index date). Among individuals treated in primary care (*n* = 37,183), 53,640 (6.6%) received at least one antithrombotic prescription at any time after the index date, of whom 37,183 (4.6%) received it within ±14 days from diagnosis, with 7505 (0.93%) being new users. In contrast, of the 78,499 subjects from hospitals, 60,743 (77.3%) had antithrombotic prescriptions at any time after COVID-19 diagnosis, 42,041 (53.5%) received it within ±14 days, and 20,300 (25.8%) were new users (Figure 1).

**Figure 3:**
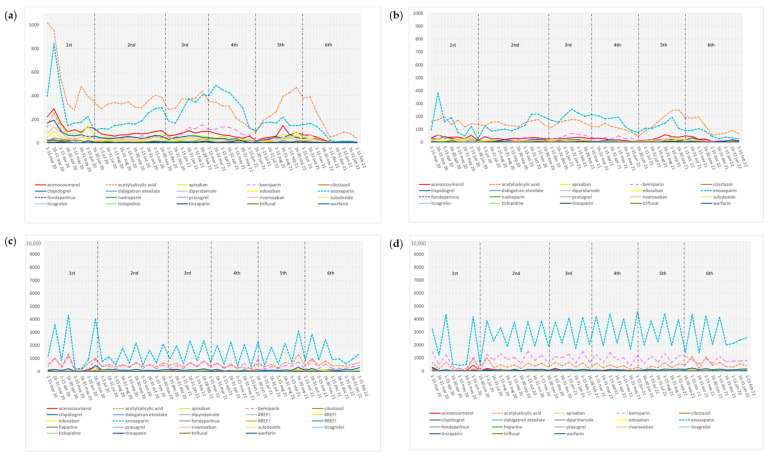
Use rates by ±14-day period in prior users (**a**) and new users (**b**) from primary care, and prior users (**c**) and new users (**d**) from hospitals.

Use rates by 14-day period were calculated per 10,000 cases. Figure 3 shows the scale we used to represent these rates. Due to significant differences, the scale for primary care patients was 1000 points, while we used a scale of 10,000 for hospitalized individuals.

The average use rates per 10,000 cases were higher in prior users than in naive users from primary care, with acetylsalicylic acid (331.3, IQR 278.8–396.8 vs. 139.9 ± 45.2, *p* = 0.000), enoxaparin (169.5, IQR 178–626.9 vs. 115.9, IQR 109.5–152.4, *p* = 0.025), acenocoumarol (70.7, IQR 66–101 vs. 29.5 ± 13.2, *p* = 0.0001), and bemiparin (48.2, IQR 47.3–76.2 vs. 22.1, IQR 18.9–28.7, *p* = 0.000) found to be the most prescribed medicines (Table 2). Despite acetylsalicylic acid being the leading prescriptions in prior users, enoxaparin was the most prescribed antithrombotic from December 2020 to June 2021 (during the third and fourth waves of COVID-19) (Figure 3).

In prior users from hospitals, the average use rates per 10,000 cases demonstrated that enoxaparin (1378, IQR 1162–1751.6), acetylsalicylic acid (486.6, IQR 480.1–655.4), and bemiparin (375, IQR 361.1–522.4) were the most prescribed antithrombotics. In new users, enoxaparin remained the most used (2348.2, IQR 2390–3123.1); however, it was followed by bemiparin (902.2 ± 368.9) and acetylsalicylic acid (390.2, IQR 372.9–529) (Table 2).

#### Post-Discharge Use

Of the subjects who received antithrombotics during admission, 6686 (15.9%) continued receiving them after discharge, but most were prior users (67.8%) (Figure 1). Although post-discharge users were mostly females (54.7%), the frequency of comorbidities at baseline was generally higher in males (Table S3).

During the 40-day follow-up period, 1438 (21.5%) patients used enoxaparin, 517 (7.7%) bemiparin, 165 (2.5%) acenocoumarol, and 107 (1.6%) acetylsalicylic acid. DOACs were poorly prescribed after discharge, as apixaban, edoxaban, and rivaroxaban were prescribed in 54 (0.8%), 33 (0.5%), and 24 (0.35%) patients, respectively. The median post-discharge use of antithrombotics in new users was ten days (IQR 9–30 days).

## 4. Discussion

The present study analyzed the antithrombotic use patterns in 882,540 COVID-19 patients from Spain. Our study found higher prescription rates of anticoagulants than antiplatelets, with higher use in hospitals than in primary care. However, the proportion of users in the wards was lower than expected. Most antithrombotic use occurred in prior users, with women accounting for most of the new users.

To the best of the authors’ knowledge, this is the first time that patterns of antithrombotic use in primary care and hospital-admitted patients with COVID-19 have been studied in Spain. Previous investigations have described their use in ICUs or across one AC [15,16]. A recent Spanish study reported the prescription rates of antithrombotics of the heparin group in pregnant women during the pandemic [17]. However, to the best of our understanding, our study is the first nationwide real-world data analysis reporting antithrombotic use rates in the general population.

Our study identified a consensus on managing COVID-19, including prescribing LWMH in hospital-admitted individuals and avoiding antithrombotics for non-hospitalized patients, as it was demonstrated that the frequency of antithrombotic use was more than ten times higher in hospitalized subjects than in the primary care population.

Nevertheless, anticoagulant prescriptions on the wards seemed lower than reported in other investigations [13,15]. For instance, a study on COVID-19 in critically ill inpatients from Spain demonstrated that 89.1% received LMWH at hospital admission [15,17]. In our research, 77.3% of inpatients received antithrombotics at any time after the index date. However, 53.6% of the hospital-admitted patients used them within ±2 weeks of diagnosis, with half of them being prior users. It must be noted that hospital-admitted patients were identified based on hospital admission or discharge; thus, since the length of hospitalization was not gathered, it is unclear whether short hospital stays of mild cases that did not always require anticoagulant therapy, some of which took place in hotels with medical supervision during the first waves of the pandemic [18,19], could have affected the use rates that we obtained.

Considering the average antithrombotic (of the heparin group) prescription rates of 26.0 and 14.6 per 1000 found in pregnant women with and without COVID-19 from Spain [17], our results demonstrated higher prescription rates for the general population. In addition, if we account for the proportion of individuals who had antithrombotic prescriptions at any time after the index date (*n* = 114,383, 12.9%), it was similar to the annual prevalence proportion of antithrombotic prescriptions (13.6%) reported in 2018 in the Spanish primary care population [20].

Although enoxaparin and bemiparin were the most prescribed in the wards, acetylsalicylic acid had an average use rate almost three times higher in new users from hospitals than their primary care counterparts. Recent studies do not support including antiplatelet therapy in the standard care of COVID-19, regardless of its severity or concomitant anticoagulation [21]. However, continuing the chronic use of antithrombotics is internationally recommended, except in the case of significant bleeding or other contraindications [8].

In primary care, antithrombotic prescription rates were low. Acetylsalicylic acid was the most prescribed, and only two out of ten prescriptions were for naive users, of whom women (67.7%) were generally younger than men (65.8% vs. 48.4% were ≤55 years old). During hospitalization, most of the new users were women (54.7%). After hospital discharge, a high frequency of females receiving antithrombotics was also found despite males generally having more comorbidities at COVID-19 diagnosis. The risk of thrombosis in COVID-19 seems higher in men than women, being more frequent in younger age groups [22]. Nonetheless, it could be possible that some of the prescriptions in antithrombotic naive women were indicated as an effort to decrease the thrombotic risks associated with contraceptive therapy, postmenopausal hormone therapy, or even pregnancy [17,23,24]. However, the medical indications for antithrombotic therapy were outside the scope of this research.

The ASH guideline panel suggests avoiding prescribing thromboprophylaxis in post-discharged patients with COVID-19 without suspected or confirmed VTE or another indication for anticoagulation, and recommends reasonable thromboprophylaxis in subjects with an increased risk of thrombosis and a low risk of bleeding [9]. Our results indicate that this guidance seems to have been adopted, as only one in every three subjects who continued using antithrombotics after discharge were new users.

Regarding baseline characteristics, it was found that in the wards, prior users were older, had more comorbidities, and used more medications before COVID-19 than new users. Interestingly, prior users from both primary care and hospitals presented an increased previous use of antidepressants and anxiolytics.

This study did not investigate the clinical outcomes associated with the use of anticoagulants. For instance, we did not study the dosage of antithrombotics or the occurrence of adverse outcomes such as COVID-19-related thrombosis or mortality; therefore, it is uncertain whether their use was preventive or therapeutic or whether using anticoagulants during hospitalization decreased the risk of death.

Nevertheless, this real-world analysis may provide evidence from antithrombotic use in the real world, which can help establish a broad picture of their place in clinical practice during the pandemic.

The strength of this study includes the use of a large number of COVID-19 diagnoses and the use of data from an established and validated database owned by the AEMPS, in which ten regions are usually involved and, as in countries with universal health care systems like the UK, the PCP is the gatekeeper of the health care system [14]. However, some limitations of the current study should also be addressed. Although the data from BIFAP represent the Spanish population concerning age, sex, and geographical region [14], only four regions provided data that can be used to perform COVID-19 studies. In BIFAP, regarding the prescriptions/dispensing issued by the PCP, the word “use” appears throughout the paper. However, we do not have complete information on actual drug intake; thus, we think this could have had a minor impact due to the types of drugs included in this study. Finally, data on COVID-19 cases pertaining to drugs prescribed by physicians other than those in the public sector are missing.

## Figures and Tables

**Table 1:** Population characteristics based on their antithrombotic use patterns.

Characteristics	All Cases	Non-Users	Users											
	*n* = 882,540	*n* = 803,306	*n* = 79,234 (9%)											
	−100%	−91%												
			Primary Care				Hospital							
			*n* = 37,183				*n* = 42,051							
											Post-Discharge			
											*n* = 6686			
			Total Users	Prior Users	Naïve Users	*p*-Value	Total Users	Prior Users	Naïve Users	*p*-Value	Total Users	Prior Users	Naïve Users	*p*-Value
			*n* = 37,183	*n* = 29,678	*n* = 7505		*n* = 42,051	*n* = 21,751	*n* = 20,300		*n* = 6686	*n* = 4531	*n* = 2155	
			(100%)	(79.8%)	(20.2%)		(100%)	(51.7%)	(48.3%)		−100%	(67.8%)	(32.2%)	
Gender														
Female	471,267 (53.4%)	433,010 (53.9%)	19,544 (52.6%)	14,463 (48.7%)	5081 (67.7%)	<0.0001	18,713 (45.5%)	9864 (45.3%)	11,451 (56.4%)	<0.0001	3659 (54.7%)	2513 (55.5%)	1146 (53.2%)	0.084
Male	411,273 (46.6%)	370,296 (46.1%)	17,639 (47.4%)	15,215 (51.3%)	2424 (32.3%)		23,338 (55.5%)	11,887 (54.7%)	8849 (45.6%)		3027 (45.3%)	2018 (44.5%)	1009 (46.8%)	
Age (y)														
Median, years (IQR)	45 (33–59)	44 (32–56)	70 (57–82)	73 (62–84)	49 (35–63)	<0.0001	70 (56–83)	78 (67–86)	60 (48–73)	<0.0001	75 (63–85)	79 (70–86)	63 (52–76)	<0.0001
18–25	100,604 (11.4%)	99,774 (12.4%)	418 (1.1%)	78 (0.2%)	340 (0.5%)		412 (0.9%)	43 (0.2%)	369 (1.8%)		24 (0.4%)	0 (0%)	24 (1.1%)	
25–40	223,369 (25.3%)	217,729 (27.1%)	3060 (8.3%)	778 (2.6%)	2282 (30.4%)		2580 (6.1%)	403 (1.9%)	2177 (10.7%)		201 (3%)	32 (0.7%)	169 (7.8%)	
40–55	275,987 (31.3%)	264,513 (32.9%)	4758 (12.8%)	2870 (9.7%)	1888 (25.2%)		6716 (16%)	1503 (6.9%)	5213 (25.7%)		638 (9.5%)	186 (4.1%)	452 (20.1%)	
55–70	163,509 (18.5%)	142,507 (17.8%)	10,128 (27.2%)	8462 (28.6%)	1666 (22.2%)		10,874 (25.9%)	4417 (20.3%)	6457 (31.8%)		1570 (23.5%)	877 (19.3%)	693 (32.2%)	
>70	119,071 (13.5%)	78783 (9.8%)	18,819 (50.6%)	17,490 (58.9%)	1329 (17.7%)		21,469 (51.1%)	15,385 (70.7%)	6084 (30%)		4253 (63.6%)	3436 (78.5%)	817 (37.9%)	
Comorbidities														
Obesity	97,000 (11%)	80,329 (10%)	7743 (20.9%)	6776 (22.8%)	967 (12.9%)	<0.0001	8928 (21.2%)	5354 (24.6%)	3574 (17.6%)	<0.0001	1664 (25%)	1172 (25.9%)	492 (22.8%)	0.0074
Cardiovascular diseases	238,176 (27%)	185,949 (23.1%)	26,491 (72.5%)	24,430 (82.3%)	2061 (27.5%)	<0.0001	25,736 (61.2%)	17,333 (79.7%)	8403 (41.4%)	<0.0001	4928 (74%)	3913 (86.3%)	1015 (47.1%)	<0.00001
Hypertension	191,354 (21.7%)	148,044 (18.4%)	21,169 (56.9%)	19,411 (65.4%)	1758 (23.4%)	<0.0001	22,141 (52.7%)	14,590 (67.1%)	7551 (37.2%)	<0.0001	4154 (62.4%)	3231 (71.3%)	923 (42.8%)	<0.00001
Heart failure	16,838 (1.9%)	9547 (1.2%)	3164 (8.5%)	3082 (10.4%)	82 (1.1%)	<0.0001	4127 (9.8%)	3725 (17.1%)	402 (2%)	<0.0001	881 (13.2%)	833 (18.4%)	48 (2.2%)	<0.00001
Arrhythmia	61,617 (7%)	45,728 (5.7%)	8758 (23.6%)	8393 (28.3%)	365 (4.9%)	<0.0001	7131 (17%)	5994 (27.6%)	1137 (5.6%)	<0.0001	1544 (23.2%)	1422 (31.4%)	122 (5.7%)	<0.00001
Ischemic heart disease	29,701 (3.4%)	17,351 (2.2%)	7673 (20.6%)	7605 (25.6%)	68 (0.9%)	<0.0001	4677 (11.1%)	4339 (20%)	338 (1.7%)	<0.0001	1236 (18.6%)	1199 (26.5%)	37 (1.7%)	<0.00001
Coronary artery disease	18,989 (2.4%)	10,011 (1.2%)	5576 (15%)	5534 (18.6%)	42 (0.6%)	<0.0001	3402 (8.1%)	3260 (15%)	142 (0.7%)	<0.0001	1017 (15.3%)	999 (22%)	18 (0.8%)	<0.00001
IAM	10,463 (1.2%)	5108 (0.6%)	3576 (9.6%)	3558 (12%)	18 (0.2%)	<0.0001	1779 (4.2%)	1719 (8%)	60 (0.3%)	<0.00001	465 (7%)	455 (10%)	10 (0.5%)	<0.00001
Valvopathies	6868 (0.8%)	4344 (0.5%)	1340 (3.6%)	1289 (4.3%)	51 (0.7%)	<0.0001	1184 (2.8%)	1026 (4.7%)	158 (0.8%)	<0.00001	247 (3.7%)	224 (5%)	23 (1.1%)	<0.00001
Peripheral arterial disease	6898 (0.8%)	3890 (0.5%)	1638 (4.4%)	1614 (5.4%)	24 (0.3%)	<0.0001	1370 (3.3%)	1252 (5.8%)	118 (0.6%)	<0.00001	308 (4.6%)	295 (6.5%)	13 (0.6%)	<0.00001
Cancer history	54,846 (6.2%)	44,097 (5.5%)	4592 (12.3%)	4178 (14.1%)	414 (5.5%)	<0.0001	6157 (14.6%)	4314 (19.8%)	1843 (9.1%)	<0.00001	993 (14.9%)	802 (17.7%)	191 (8.9%)	<0.00001
Hereditary thrombophilia	654 (0.1%)	503 (0.1%)	111 (0.3%)	88 (0.3%)	23 (0.3%)	0.876	40 (0.1%)	34 (0.2%)	6 (0.03%)	<0.00001	13 (0.2%)	13 (0.3%)	0 (0%)	0.052
Stroke	20,382 (2.3%)	10,962 (1.4%)	5615 (15.1%)	5555 (18.7%)	60 (0.8%)	<0.0001	3805 (9%)	3577 (16.4%)	228 (1.1%)	<0.00001	958 (14.3%)	920 (20.3%)	38 (1.8%)	<0.00001
Ischemic stroke	18,967 (2.1%)	9893 (1.2%)	5471 (14.7%)	5420 (18.3%)	51 (0.7%)	<0.0001	3603 (8.6%)	3433 (15.8%)	170 (0.8%)	<0.00001	900 (13.5%)	870 (19.2%)	30 (1.4%)	<0.00001
Hemorrhagic stroke	2095 (0.2%)	1483 (0.2%)	287 (0.8%)	277 (0.9%)	10 (0.1%)	<0.0001	325 (0.8%)	256 (1.2%)	69 (0.3%)	<0.00001	58 (0.9%)	49 (1%)	9 (0.4%)	0.006
Acute liver disease	103 (0.0%)	85 (0.0%)	9 (0.02%)	8 (0.03%)	1 (0.01%)	0.499	9 (0.02%)	5 (0.02%)	4 (0.02%)	0.8181	0 (0%)	0 (0%)	0 (0%)	NA
CKD	18,317 (2.1%)	11,633 (1.4%)	2891 (7.8%)	2780 (9.4%)	111 (1.5%)	<0.0001	3793 (9%)	3117 (14.3%)	676 (3.3%)	<0.00001	762 (11.4%)	675 (14.9%)	87 (4%)	<0.00001
COPD	17,962 (2%)	12,164 (1.5%)	2209 (5.9%)	2064 (6.7%)	145 (1.9%)	<0.0001	3589 (8.5%)	2687 (12.4%)	902 (4.4%)	<0.00001	676 (10.2%)	570 (12.6%)	106 (4.9%)	<0.00001
Asthma	77,974 (8.8%)	71,307 (8.9%)	3029 (8.2%)	2340 (7.9%)	689 (9.2%)	0.0002	3638 (8.7%)	2012 (9.2%)	1626 (8%)	<0.00001	595 (8.9%)	436 (9.6%)	159 (7.4%)	0.0026
Multiple sclerosis	1744 (0.2%)	1565 (0.2%)	70 (0.2%)	51 (0.2%)	19 (0.3%)	0.143	109 (0.3%)	54 (0.2%)	55 (0.3%)	0.645	13 (0.2%)	12 (0.3%)	1 (0.004%)	0.587
Parkinson’s disease	4125 (0.5%)	2605 (0.3%)	658 (1.8%)	608 (2%)	50 (0.7%)	<0.0001	862 (2%)	654 (3%)	208 (1%)	<0.00001	153 (2.3%)	131 (2.9%)	22 (1%)	<0.00001
Dementia	10,598 (1.2%)	6806 (0.8%)	1870 (5%)	1718 (5.8%)	152 (2%)	<0.0001	1922 (4.6%)	1502 (6.9%)	420 (2%)	<0.00001	330 (5%)	283 (6.2%)	47 (2.2%)	<0.00001
Alzheimer’s disease	5453 (0.6%)	3604 (0.4%)	901 (2.4%)	806 (2.7%)	95 (1.3%)	<0.0001	948 (2.3%)	712 (3.3%)	236 (1.2%)	<0.0001	159 (2.4%)	133 (2.9%)	26 (1.2%)	<0.00001
IBD	5764 (0.7%)	5196 (0.6%)	254 (0.7%)	199 (0.7%)	55 (0.7%)	0.543	314 (0.7%)	172 (0.8%)	142 (0.7%)	0.276	41 (0.6%)	30 (0.7%)	11 (0.5%)	0.459

AMI: acute myocardial infarction; CKD: chronic kidney disease; COPD: chronic obstructive pulmonary disease; IBD: inflammatory bowel disease.

**Table 2:** Median antithrombotic prescription rates per 10,000 cases between 2020 and 2022 in primary care and hospitals.

	Study Cohort				Hospitalized Population			Primary Care Population		
	*n* = 882,540				*n* = 78,499			*n* = 804,041		
Antithrombotic	Global Use	Total Use in Hospitals	Total Use in Primary Care	*p* Value	New Users	Prior Users	*p* Value	New Users	Prior Users	*p* Value
Acenocoumarol	130.6 (125.7–161.3)	147.1(143.7–198.8)	111.2 (92.8–133.4)	0.000	66.6 (62.6–102.2)	69.3 (69–103.8)	0.838	29.5 ± 13.2	70.7 (66–101)	0.000
Acetylsalicylic acid	723 (670.1–839.9)	973.4 (905–1132.5)	466.3 (410.6–544.9)	0.000	390.2 (372.9–529)	486.6 (480.1–655.4)	0.307	139.9 ± 45.2	331.3 (278.8–396.8)	0.000
Apixaban	102.5 (93.3–122.5)	121.6 (129–160.8)	61.4 (58.3–88.6)	0.000	47.2 (43.9–68.6)	67.1 (66.9–100.2)	0.066	15.7 ± 9.7	46.9 (44.4–71.1)	0.000
Bemiparin	694.8 (583.8–860.8)	1341.3 (1242.3–1445.6)	69.8 (67.3–103.9)	0.000	902.2 ± 368.9	375 (361.1–522.4)	0.000	22.1 (18.9–28.7)	48.2 (47.3–76.2)	0.000
Cilostazol	6.4 (6–11.6)	0.0	8.4 (6.9–15.5)	0.008	0.0	4.5 (2–7)	0.020	1.5 (1.3–2.4)	5.8 (5–13.6)	0.000
Clopidogrel	141.8 (131.6–180.8)	229 (212.5–274.9)	56.7 (53–78.5)	0.000	105 (89.6–122.7)	119.5 (110.4–164.6)	0.838	14.6 (13–17.9)	42.4 (38.5–62)	0.000
Dabigatran	19.6 (19–29.5)	26.4 (24.3–40.4)	16.5 (14.3–20.2)	0.002	8.4 (7.4–17.3)	17 (13.3–26.6)	0.307	4.1 (3.2–4.9)	11.7 (10.1–16.2)	0.000
Dalteparin	0.0	0.0	0.0	0.066	0.0	0.0	0.561	0.0	0.0	0.674
Dipyridamole	0.0	0.0	0.0	0.340	0.0	0.0	0.991	0.0	0.0	0.240
Edoxaban	35.7 (35–51.6)	44 (39.5–66.2)	32 (28.4–40.9)	0.066	17.8 (15.3–32.2)	20 (18.4–39.7)	0.838	7.9 (7.3–10.5)	24.3 (20.4–30.9)	0.000
Enoxaparin	750 (1888.8–2727)	4408.5 (3934.4–4492.7)	297.1 (291.4–418.3)	0.000	2348.2 (2390–3123.1)	1378 (1162–1751.6)	0.002	115.9 (109.5–152.4)	169.5 (178–626.9)	0.025
Fondaparinux	8 (8.7–18)	19.8 (18.3–33.7)	0.0	0.000	13.7 (10.5–21.4)	8.4 (6.2–13.9)	0.153	0.0	0.0	0.233
Heparin	6.5 (6.3–13)	19.9 (14.2–19.1)	0.0	0.000	8.1 (8–19)	5.3 (3.8–8.3)	0.025	0.0	0.0	0.516
Iloprost	0.0	0.0	0.0	0.125	0.0	0.0	0.241	0.0	0.0	0.716
Nadroparin	0.0	0.0	0.0	0.326	0.0	0.0	0.998	0.0	0.0	0.225
Prasugrel	0.0	0.0	0.0	0.301	0.0	0.0	0.808	0.0	0.0	0.831
Rivaroxaban	49.5 (49.2–63.4)	56.6 (54.5–76.8)	40 (37.6–54.3)	0.008	25.6 (22.8–42.4)	32 (26.8–39.2)	0.307	11.9 (10.8–16.4)	29.4 (25.4–39.1)	0.000
Selexipag	0.0	0.0	0.0	0.125	0.0	0.0	0.475	0.0	0.0	0.479
Sulodexide	2.8 (2–4.9)	0.0	6 (5.7–8.6)	0.000	0.0	0.0	0.475	1.6 (1.5–3.1)	3.8 (3.5–6.1)	0.008
Ticagrelor	12.1 (13.1–22)	22.7 (17.4–34)	7.9 (7.6–10.9)	0.000	7.7 (6.7–15)	11.8 (9.4–20.2)	0.117	1.9 (1.6–3.4)	6.0 (5.4–8.0)	0.000
Ticlopidine	0.0	0.0	0.0	0.125	0.0	0.0	0.125	0.0	0.0	0.109
Tinzaparin	2.2 (2.1–4.1)	0.0	2.9 (2.9–5.7)	0.040	0.0	0.0	0.522	0.0	2.7 (2.7–5.5)	0.000
Triflusal	9.4 (9.3–14.1)	10.5 (9.8–17.7)	8.2 (7–12.1)	0.307	0.0	6.4 (5.4–12.8)	0.066	2.4 (1.9–3.5)	5.8 (4.4–9.1)	0.002
Warfarin	3 (2.4–8.4)	0.0	4 (3.2–5.7)	0.066	0.0	0.0	0.999	1.1 (0.8–1.8)	2.4 (2.2–4.1)	0.008

## Data Availability

Individual data from BIFAP cannot be made publicly available. For the dataset, BIFAP can be contacted at the following email address: bifap@aemps.es.

## Supplementary Materials

The following supporting information can be downloaded at: https://www.mdpi.com/article/10.3390/jcm13082403/s1, Table S1: Antthrombotic agents, ATC codes; Table S2: Population characteristics based on their antithrombotic use patterns.; Table S3: Medications prescribed within six months before COVID-19 diagnosis; Table S4: Baseline characteristics by gender.
