# Supplementary material for: Antithrombotic Use Patterns in COVID-19 Patients from Spain: A Real-World Data Study
Source: J Clin Med. 2024 Apr 20;13(8):2403. doi: 10.3390/jcm13082403 (PMC11051525; doi:10.3390/jcm13082403)
Supplement: Supplementary file 1 [file jcm-13-02403-s001.zip › jcm-2917038-supplementary.pdf]

Table S1. Antthrombotic agents, ATC codes

B01AA Vitamin K antagonists

ATC code	Name
B01AA01	dicoumarol
B01AA02	phenindione
B01AA03	warfarin
B01AA04	phenprocoumon
B01AA07	acenocoumarol
B01AA08	ethyl biscoumacetate
B01AA09	clorindione
B01AA10	diphenadione
B01AA11	tioclomarol
B01AA12	fluindione

B01AB Heparin group

ATC code	Name
B01AB01	heparin
B01AB02	antithrombin III
B01AB04	dalteparin
B01AB05	enoxaparin
B01AB06	nadroparin
B01AB07	parnaparin
B01AB08	reviparin
B01AB09	danaparoid
B01AB10	tinzaparin
B01AB11	sulodexide
B01AB12	bemiparin
B01AB51	heparin, combinations

B01AC Platelet aggregation inhibitors excl. heparin

ATC code	Name
B01AC01	ditazole
B01AC02	cloricromen
B01AC03	picotamide
B01AC04	clopidogrel
B01AC05	ticlopidine
B01AC06	acetylsalicylic acid
B01AC07	dipyridamole
B01AC08	carbasalate calcium
B01AC09	epoprostenol
B01AC10	indobufen
B01AC11	iloprost
B01AC13	abciximab
B01AC15	aloxiprin
B01AC16	eptifibatide
B01AC17	tirofiban

TABLE S2. Population characteristics based on their antithrombotic use patterns.

Characteristics	Users* n=79,234 (9%)		
	Primary care n=37,183	Hospital n=42,051	p value
**Gender**			
Female	19 544 (52.6%)	18 713 (45.5%)	< 0.001
Male	17 639 (47.4%)	23 338 (55.5%)	
**Age (y)**			
Median, years (IQR)	70 (57-82)	70 (56-83)	
18-25	418 (1.1%)	412 (0.9%)	
25-40	3 060 (8.3%)	2 580 (6.1%)	
40-55	4 758 (12.8%)	6 716 (16%)	< 0.001
55-70	10 128 (27.2%)	10 874 (25.9%)	
>70	18 819 (50.6%)	21 469 (51.1%)	
**Comorbidities**			
Obesity	7 743 (20.9%)	8 928 (21.2%)	0.160
Cardiovascular diseases	26 491 (72.5%)	25 736 (61.2%)	< 0.001
Hypertension	21 169 (56.9%)	22 141 (52.7%)	< 0.001
Heart failure	3 164 (8.5%)	4 127 (9.8%)	< 0.001
Arrhythmia	8 758 (23.6%)	7 131 (17%)	< 0.001
Ischemic heart disease	7 673 (20.6%)	4 677 (11.1%)	< 0.001
Coronary artery disease	5 576 (15%)	3 402 (8.1%)	< 0.001
IAM	3 576 (9.6%)	1 779 (4.2%)	< 0.001
Valvopathies	1 340 (3.6%)	1 184 (2.8%)	< 0.001
Peripheral arterial disease	1 638 (4.4%)	1 370 (3.3%)	< 0.001
Cancer history	4 592 (12.3%)	6 157 (14.6%)	< 0.001
Hereditary thrombophilia	111 (0.3%)	40 (0.1%)	< 0.001
Stroke	5 615 (15.1%)	3 805 (9%)	< 0.001
Ischemic stroke	5 471 (14.7%)	3 603 (8.6%)	< 0.001
Hemorrhagic stroke	287 (0.8%)	325 (0.8%)	0.987
Acute liver disease	9 (0.02%)	9 (0.02%)	0.794

Table S3.Medications prescribed within six months before COVID-19 diagnosis

Medicines	All cases n=882,540 (100 %)	Non-users n=803,306 (91 %)	Primary care users. Global users n=37,183 (4,2%)	Prevalent users n=29,678 (%)	Incident users n=7,505 (%)	Hospitals users Global users n=42,051 (4,8%)	Prevalent users n=21,751 (%)	Incident users n=20,300 (%)
A10 Drugs used in diabetes	60 308 (6.8%)	42 107 (5.2%)	9.040 (24.3%)	8.594 (29.0%)	446 (5.9%)	9.161 (21.8%)	6.703 (30.8%)	2.458(12.1%)
A10A Insulins and analogues	17 812 (2.0%)	11 355 (1.4%)	2.915 (7.8%)	2.812 (9.5%)	103 (1.4%)	3.542 (8.4%)	2.905 (13.4%)	637 (3.1%)
A10B Blood glucose lowering drugs, excluding insulins	53 414 (6.1%)	37 245 (4.6%)	8.205 (22.1%)	7.806 (26.3%)	399 (5.3%)	7.964 (18.9%)	5.723 (26.3%)	2.241 (11.0%)
C01B Antiarrhythmics, class I and III	4 281 (0.5%)	2.419 (0.3%)	1.049 (2.8%)	1.040 (3.5%)	9 (0.1%)	813 (1.9%)	763 (3.5%)	50 (0.2%)
C02 Antihypertensives	7 452 (0.8%)	4.624 (0.6%)	1.157 (3.1%)	1.106 (3.7%)	51 (0.7%)	1.671 (4.0%)	1.311 (6.0%)	360 (1.8%)
C02C Antiadrenergic agents, peripherally acting	6 930 (0.8%)	4.247 (0.5%)	1.084 (2.9%)	1.039 (3.5%)	45 (0.6%)	1.599 (3.8%)	1.256 (5.8%)	343 (1.7%)
C03 Diuretics	50 612 (57%)	30.852 (3.8%)	8.424 (22.7%)	8.046 (27.1%)	378 (5.0%)	11.336(27.0%)	9.206 (42.3%)	2.130 (10.5%)
C03A Low-ceiling diuretics, thiazides	5 605 (0.6%)	3.846 (0.5%)	649 (1.7%)	603 (2.0%)	46 (0.6%)	1.110 (2.6%)	807 (3.7%)	303 (1.5%)
C03B Low-ceiling diuretics, excluding thiazides	4 476 (0.5%)	3.344 (0.4%)	509 (1.4%)	455 (1.5%)	54 (0.7%)	623 (1.5%)	409 (1.9%)	214 (1.1%)
C03C High-ceiling diuretics	38 057 (4.3%)	21.520 (2.7%)	6.775 (18.2%)	6.530 (22.0%)	245 (3.3%)	9.762 (23.2%)	8.241 (37.9%)	1.521 (7.5%)
C03D Aldosterone antagonists and other potassium-sparing agents	7 656 (0.9%)	4.290 (0.5%)	1.573 (4.2%)	1.546 (5.2%)	27 (0.4%)	1.793 (4.3%)	1.624 (7.5%)	169 (0.8%)
C03E Diuretics and potassium-sparing agents in combination	2 559 (0.3%)	1.871 (0.2%)	319 (0.9%)	282 (1.0%)	37 (0.5%)	369 (0.9%)	233 (1.1%)	136 (0.7%)
C07 Beta blocking agents	48 225 (5.5%)	30.167 (3.8%)	10.318(27.7%)	10.098(34.0%)	220 (2.9%)	7.740 (18.4%)	6.670 (30.7%)	1.070 (5.3%)
C07A Beta blocking agents	46 821 (5.3%)	29.072 (3.6%)	10.130 (27.2%)	9.923 (33.4%)	207 (2.8%)	7.619 (18.1%)	6.597 (30.3%)	1.022 (5.0%)
C07B Beta blocking agents and thiazides	1 028 (0.1%)	795 (0.1%)	128 (0.3%)	119 (0.4%)	9 (0.1%)	105 (0.2%)	66 (0.3%)	39 (0.2%)
C08 Calcium channel blockers	30 323 (3.4%)	19.734 (2.5%)	4.758 (12.8%)	4.548 (15.3%)	210 (2.8%)	5.831 (13.9%)	4.469 (20.5%)	1.362 (6.7%)
C08C Selective calcium channel blockers with mainly vascular effects	26 856 (3.0%)	17.714 (2.2%)	3.947 (10.6%)	3.745 (12.6%)	202 (2.7%)	5.195 (12.4%)	3.899 (17.9%)	1.296 (6.4%)
C08D Selective calcium channel blockers with direct cardiac effects	3 717 (0.4%)	2.130 (0.3%)	866 (2.3%)	857 (2.9%)	9 (0.1%)	721 (1.7%)	654 (3.0%)	67 (0.3%)
C09 Agents acting on the renin-angiotensin system	126 782(14.4%)	94.361 (11.7%)	16.337 (43.9%)	15.289 (51.5%)	1.048(14.0%)	16.084 (38.2%)	10.918(50.2%)	5.166 (25.4%)

C09A ACE inhibitors, plain	39 155 (4.4%)	28.161 (3.5%)	5.502 (14.8%)	5.167 (17.4%)	335 (4.5%)	5.492 (13.1%)	3.987 (18.3%)	1.505 (7.4%)
C09B ACE inhibitors, combinations	13 761 (1.6%)	10.728 (1.3%)	1.466 (3.9%)	1.329 (4.5%)	137 (1.8%)	1.567 (3.7%)	902 (4.1%)	665 (3.3%)
C09C Angiotensin II receptor blockers (ARBs), plain	39 587 (4.5%)	28.894 (3.6%)	4.864 (13.1%)	4.565 (15.4%)	299 (4.0%)	5.829 (13.9%)	4.095 (18.8%)	1.734 (8.5%)
C09D Angiotensin II receptor blockers (ARBs), combinations	42 218 (4.8%)	31.549 (3.9%)	5.456 (14.7%)	5.122 (17.3%)	334 (4.5%)	5.213 (12.4%)	3.465 (15.9%)	1.748 (8.6%)
C10 Lipid modifying agents	126 883(14.4%)	94.811 (11.8%)	17.696 (47.6%)	16.763 (56.5%)	933 (12.4%)	14.376 (34.2%)	10.200 (46.9%)	4.176 (20.6%)
C10A Lipid modifying agents, plain	114 481(13.0%)	85.677 (10.7%)	15.555 (41.8%)	14.704 (49.5%)	851 (11.3%)	13.249 (31.5%)	9.394 (43.2%)	3.855 (19.0%)
C10AA HMG CoA reductase inhibitors	103 737(11.8%)	76.997 (9.6%)	14.513 (39.0%)	13.769 (46.4%)	744 (9.9%)	12.227 (29.1%)	8.805 (40.5%)	3.422 (16.9%)
C10B Lipid modifying agents, combinations	15 103 (1.7%)	10.879 (1.4%)	2.648 (7.1%)	2.557 (8.6%)	91 (1.2%)	1.576 (3.7%)	1.189 (5.5%)	387 (1.9%)
G03 Sex hormones and modulators of the genital system	35 355 (4.0%)	34.338 (4.3%)	509 (1.4%)	235 (0.8%)	274 (3.7%)	508 (1.2%)	148 (0.7%)	360 (1.8%)
G03A Hormonal contraceptives for systemic use	28 919 (3.3%)	28.450 (3.5%)	208 (0.6%)	45 (0.2%)	163 (2.2%)	261 (0.6%)	44 (0.2%)	217 (1.1%)
G03C Estrogens	1 076 (0.1%)	1.006 (0.1%)	33 (0.1%)	23 (0.1%)	10 (0.1%)	37 (0.1%)	14 (0.1%)	23 (0.1%)
H02 Corticosteroids for systemic use	44 826 (5.1%)	31.843 (4.0%)	3.078 (8.3%)	2.576 (8.7%)	502 (6.7%)	9.905 (23.6%)	6.181 (28.4%)	3.724 (18.3%)
H02A Corticosteroids for systemic use, plain	44.826 (5.1%)	31.843 (4.0%)	3.078 (8.3%)	2.576 (8.7%)	502 (6.7%)	9.905 (23.6%)	6.181 (28.4%)	3.724 (18.3%)
H02AB Glucocorticoids	44 783 (5.1%)	31.814 (4.0%)	3.072 (8.3%)	2.570 (8.7%)	502 (6.7%)	9.897 (23.5%)	6.177 (28.4%)	3.720 (18.3%)
J01 Antibacterials for systemic use	156 686(17.8%)	128.371(16.0%)	10.337 (27.8%)	8.492 (28.6%)	1.845 (24.6%)	17.978 (42.8%)	11.347 (52.2%)	6.631 (32.7%)
J01C Beta-lactam antibacterials, penicillins	66 454 (7.5%)	55.560 (6.9%)	3.936 (10.6%)	3.229 (10.9%)	707 (9.4%)	6.958 (16.5%)	4.760 (21.9%)	2.198 (10.8%)
J01D Other beta-lactam antibacterials	34 531 (3.9%)	23.189 (2.9%)	2.760 (7.4%)	2.397 (8.1%)	363 (4.8%)	8.582 (20.4%)	5.755 (26.5%)	2.827 (13.9%)
J01E Sulfonamides and trimethoprim	3 580 (0.4%)	2.378 (0.3%)	366 (1.0%)	334 (1.1%)	32 (0.4%)	836 (2.0%)	689 (3.2%)	147 (0.7%)
J01F Macrolides, lincosamides and streptogramins	43 404 (4.9%)	34.516 (4.3%)	2.786 (7.5%)	2.179 (7.3%)	607 (8.1%)	6.102 (14.5%)	3.408 (15.7%)	2.694 (13.3%)
J01G Aminoglycoside antibacterials	878 (0.1%)	538 (0.1%)	74 (0.2%)	66 (0.2%)	8 (0.1%)	266 (0.6%)	232 (1.1%)	34 (0.2%)
J01M Quinolone antibacterials	24 953 (2.8%)	17.661 (2.2%)	2.329 (6.3%)	2.088 (7.0%)	241 (3.2%)	4.963 (11.8%)	3.606 (16.6%)	1.357 (6.7%)
J01R Combinations of antibacterials	623 (0.1%)	568 (0.1%)	27 (0.1%)	22 (0.1%)	5 (0.1%)	28 (0.1%)	16 (0.1%)	12 (0.1%)
J01X Other antibacterials	34 403 (3.9%)	28.244 (3.5%)	2.572 (6.9%)	2.116 (7.1%)	456 (6.1%)	3.587 (8.5%)	2.629 (12.1%)	958 (4.7%)
J05 Antivirals for systemic use	8 847 (1.0%)	6.287 (0.8%)	499 (1.3%)	413 (1.4%)	86 (1.1%)	2.061 (4.9%)	1.063 (4.9%)	998 (4.9%)
J05A Direct-acting antiviral drugs	8 847 (1.0%)	6.287 (0.8%)	499 (1.3%)	413 (1.4%)	86 (1.1%)	2.061 (4.9%)	1.063 (4.9%)	998 (4.9%)
L Antineoplastic and immunomodulatory agents	23 305 (2.6%)	17.459 (2.2%)	1.840 (4.9%)	1.607 (5.4%)	233 (3.1%)	4.006 (9.5%)	2.513 (11.6%)	1.493 (7.4%)

L01 Antineoplastic agents	4 136 (0.5%)	2.683 (0.3%)	434 (1.2%)	400 (1.3%)	34 (0.5%)	1.019 (2.4%)	786 (3.6%)	233 (1.1%)
L01X Other antineoplastic agents	2 451 (0.3%)	1.519 (0.2%)	302 (0.8%)	283 (1.0%)	19 (0.3%)	630 (1.5%)	501 (2.3%)	129 (0.6%)
L01XX05 Hydroxycarbamide/Hydroxyurea	443 (0.1%)	235 (0.0%)	127 (0.3%)	125 (0.4%)	2 (0.0%)	81 (0.2%)	78 (0.4%)	3 (0.0%)
L02 Endocrine therapy	5 834 (0.7%)	4.403 (0.5%)	556 (1.5%)	500 (1.7%)	56 (0.7%)	875 (2.1%)	653 (3.0%)	222 (1.1%)
L02A Hormones and related agents	2720 (0.3%)	1.808 (0.2%)	337 (0.9%)	312 (1.1%)	25 (0.3%)	575 (1.4%)	449 (2.1%)	126 (0.6%)
L02B Hormone antagonists and related agents	3 803 (0.4%)	3.082 (0.4%)	292 (0.8%)	252 (0.8%)	40 (0.5%)	429 (1.0%)	299 (1.4%)	130 (0.6%)
L03A Immunostimulants	1 639 (0.2%)	1.077 (0.1%)	84 (0.2%)	74 (0.2%)	10 (0.1%)	478 (1.1%)	323 (1.5%)	155 (0.8%)
L04A Immunosuppressants	13 155 (1.5%)	10.186 (1.3%)	867 (2.3%)	721 (2.4%)	146 (1.9%)	2.102 (5.0%)	1.105 (5.1%)	997 (4.9%)
M01 Anti-inflammatory and antirheumatic products	170 874(19.4%)	155.790 (19.4%)	6.652 (17.9%)	5.168 (17.4%)	1.484 (19.8%)	8.432 (20.1%)	4.111 (18.9%)	4.321 (21.3%)
M01A Anti-inflammatory and antirheumatic products, non-steroids	169 812(19.2%)	154.874 (19.3%)	6.578 (17.7%)	5.101 (17.2%)	1.477 (19.7%)	8.360 (19.9%)	4.067 (18.7%)	4.293 (21.1%)
N03A Antiepileptics	39 658 (4.5%)	31.077 (3.9%)	3.679 (9.9%)	3.371 (11.4%)	308 (4.1%)	4.902 (11.7%)	3.493 (16.1%)	1.409 (6.9%)
N05 Psycholeptics	158 162(17.9%)	127.359 (15.9%)	13.500 (36.3%)	12.083 (40.7%)	1.417 (18.9%)	17.303 (41.1%)	11.575 (53.2%)	5.728 (28.2%)
N05A Antipsychotics	38 386 (4.3%)	28.667 (3.6%)	3.754 (10.1%)	3.352 (11.3%)	402 (5.4%)	5.965 (14.2%)	4.324 (19.9%)	1.641 (8.1%)
N05B Anxiolytics	118 541(13.4%)	96.771 (12.0%)	9.539 (25.7%)	8.522 (28.7%)	1.017 (13.6%)	12.231 (29.1%)	8.098 (37.2%)	4.133 (20.4%)
N05C Hypnotics and sedatives	32 824 (3.7%)	25.035 (3.1%)	3.220 (8.7%)	2.940 (9.9%)	280 (3.7%)	4.569 (10.9%)	3.250 (14.9%)	1.319 (6.5%)
N06 Psychoanaleptics	89 785 (10.2%)	72.612 (9.0%)	8.011 (21.5%)	7.240 (24.4%)	771 (10.3%)	9.162 (21.8%)	6.456 (29.7%)	2.706 (13.3%)
N06A Antidepressants	89 785 (10.2%)	72.612 (9.0%)	8.011 (21.5%)	7.240 (24.4%)	771 (10.3%)	9.162 (21.8%)	6.456 (29.7%)	2.706 (13.3%)
R03 Drugs for obstructive airway diseases	67 622 (7.7%)	52.928 (6.6%)	5.184 (13.9%)	4.525 (15.2%)	659 (8.8%)	9.510 (22.6%)	6.288 (28.9%)	3.222 (15.9%)
R03A Adrenergisc, inhalants	51 322 (5.8%)	40.259 (5.0%)	3.766 (10.1%)	3.242 (10.9%)	524 (7.0%)	7.297 (17.4%)	4.828 (22.2%)	2.469 (12.2%)
R03B Other drugs for obstructive airway diseases, inhalants	19 887 (2.3%)	12.819 (1.6%)	1.920 (5.2%)	1.740 (5.9%)	180 (2.4%)	5.148 (12.2%)	3.699 (17.0%)	1.449 (7.1%)
R03D Other systemic drugs for obstructive airway diseases	16 508 (1.9%)	13.396 (1.7%)	1.187 (3.2%)	1.065 (3.6%)	122 (1.6%)	1.925 (4.6%)	1.268 (5.8%)	657 (3.2%)

Table S4. Baseline characteristics by gender

					COVID-19 ANTITHROMBOTIC USERS n=79,234 (9%)													
	ALL CASES n=882,540 (100%)		*COVID-19 NON-USERS n=803,306 (91%)*		*PRIMARY CARE - GLOBAL n=37,183 (46,9%)*		*PRIMARY CARE – PRIOR USERS n=29,678 (79,8%)*		*PRIMARY CARE – NEW USERS n=7,505 (20,2%)*		*HOSPITAL - GLOBAL n=42,051 (53,1%)*		*HOSPITAL – PRIOR USERS n=21,751 (51,7%)*		*HOSPITAL – NEW USERS n=20,300 (48,3%)*		*POSTDISCHARGE USERS n=6,686 (0,2%)*	
CHARACTERISTICS	***MALE n=411,273 (46,6%)***	***FEMALE n=471,267 (53,4&)***	***MALE n=370,296 (46,1%)***	***FEMALE n=433,010 (53,9%)***	***MALE n=17,639 (47,4%)***	***FEMALE n=19,544 (52,6%)***	***MALE n=15,215 (51,3%)***	***FEMALE n=14,463 (48,7%)***	***MALE n=2424 (32,3%)***	***FEMALE n=5081 (67,7%)***	***MALE n=23,338 (55,5%)***	***FEMALE n=18,713 (45,5%)***	***MALE n=11,887 (54,7%)***	***FEMALE n=9,864 (45,3%)***	***MALE 8,849 (45,6%)***	***FEMALE 11,451 (56,4%)***	***MALE n=3,027 (45,3%)***	***FEMALE n=3,659 (54,7%)***
**Age (y)**																		
Mean/median	46.9 / 45 years	47.9 / 46 years	44.6 / 43 years	46.0 / 44 years	67.6 / 68.0 years	67.8 / 73 years	69.5 / 70.0 years	74.5 / 78 years	55.6 / 55.0 years	48.4 / 43 years	66.9 / 68.0 years	69.4 / 73 years	74.3 / 76.0 years	77.1 / 81 years	59.3 / 59.0 years	60.8 / 61	70.9 / 73.0 years	74.8 / 79 years
18-25	49155 (12%)	51449 (10.9%)	48890 (13.2%)	50884 (11.8%)	98 (0.6%)	320 (1.6%)	32 (0.2%)	46 (0.3%)	66 (3%)	274 (5.4%)	167 (0.7%)	245 (1.3%)	15 (0.1%)	28 (0.3%)	217 (2.5%)	152 (1.3%)	17 (0.6%)	7 (0.2%)
25-40	103653 (25.2%)	119716 (25.4%)	101948 (27.5%)	115781 (26.7%)	474 (2.7%)	2586 (13.2%)	172 (1.1%)	606 (4%)	302 (12.4%)	1980 (39%)	1231 (5.3%)	1349 (7.2%)	154 (1.3%)	249 (2.5%)	1100 (12.4%)	1077 (9.4%)	107 (3.5%)	94 (2.6%)
40-55	127619 (31%)	148368 (31.5%)	121001 (32.7%)	143512 (33.1%)	2518 (14.3%)	2240 (11.5%)	1718 (11.3%)	1152 (7.6%)	800 (33%)	1088 (21.4%)	4100 (17.6%)	2616 (14%)	861 (7.2%)	642 (6.5%)	1974 (22.3%)	3239 (28.3%)	246 (8.1%)	392 (10.7%)
55-70	80026 (19.5%)	83483 (17.7%)	66776 (18%)	75731 (17.5%)	6458 (36.6%)	3670 (18.8%)	5665 (37.2%)	2797 (18.4%)	793 (33%)	873 (17.2%)	6792 (29.1%)	4082 (21.8%)	2857 (24%)	1560 (15.8%)	2522 (28.5%)	3935 (34.4%)	526 (17.4%)	1044 (28.5%)
>70	50820 (12.4%)	68251 (14.5%)	31681 (8.6%)	47102 (10.9%)	8091 (45.9%)	10728 (54.9%)	7628 (50.1%)	9862 (64.8%)	463 (19.1%)	866 (17%)	11048 (47.3%)	10421 (55.7%)	8000 (67.3%)	7385 (74.9%)	3036 (34.3%)	3048 (26.6%)	2131 (70.4%)	2122 (58%)
**Comorbidities**																		
Obesity	40.076 (9.7%)	56.924 (12.1%)	32.110 (8.7%)	48.219 (11.1%)	3.660 (20,7%)	4.083 (23,1%)	3293 (21,6%)	3483 (24,1%)	367 (15,1%)	600 (11,8%)	4309 (18,5%)	4619 (24,7%)	4309 (36,2%)	2758 (28,5%)	1713 (19,4%)	1861 (21%)	829 (27,4%)	835 (22,8%)
Cardiovascular diseases	117.895 (28.7%)	120.281 (25.5%)	89.889 (24.3%)	96.060 (22.2%)	13.883 (78,7%)	12.608 (71,5%)	13034 (85,7%)	11396 (78,8%)	849 (35%)	1212 (23,9%)	14123 (60,5%)	11613 (62,1%)	9527 (80,1%)	7806 (80,6%)	4596 (51,9%)	3807 (43%)	2710 (89,5%)	2218 (60,6%)
Hypertension	93.482 (22.7%)	97.872 (20.8%)	71.225 (19.2%)	76.819 (17.7%)	10.471 (59,4%)	10.698 (60,6%)	9743 (64%)	9668 (66,8%)	728 (30%)	1030 (20,3%)	11786 (50,5%)	10355 (55,3%)	1178 (9,9%)	6936 (71,6%)	4132 (46,7%)	3419 (38,6%)	2184 (72,2%)	1970 (53,8%)
Heart insufficiency	7.601 (1.8%)	9.237 (2%)	4.160 (1.1%)	5.387 (1.2%)	1.337 (7,6%)	1.827 (10,4%)	1304 (8,6%)	1778 (12,3%)	33 (1,4%)	49 (1%)	2104 (9%)	2023 (10,8%)	2104 (17,7%)	1807 (18,7%)	186 (2,1%)	216 (2,4%)	439 (14,5%)	442 (12,1%)
Arrythmia	29.665 (7.2%)	31.952 (6.8%)	21.361 (5.8%)	24.367 (5.6%)	4.330 (24,5%)	4.428 (25,1%)	4189 (27,5%)	4204 (29,1%)	141 (5,8%)	224 (4,4%)	3974 (17%)	3157 (16,9%)	3974 (33,4%)	2628 (27,1%)	608 (6,9%)	529 (6%)	868 (28,7%)	676 (18,5%)
Ischemic heart disease	18.936 (4.6%)	10.765 (2.3%)	10.446 (2.8%)	6.905 (1.6%)	5.312 (30,1%)	2.361 (13,4%)	5282 (34,7%)	2323 (16,1%)	30 (1,2%)	38 (0,7%)	3178 (13,6%)	1499 (8%)	3178 (26,7%)	1349 (13,9%)	188 (2,1%)	150 (1,7%)	853 (28,2%)	383 (10,5%)
Coronary artery disease	12.435 (3%)	6.554 (1.4%)	6.326 (1.7%)	3.685 (0.9%)	3.794 (21,5%)	1.782 (10,1%)	3775 (24,8%)	1759 (12,2%)	19 (0,8%)	23 (0,5%)	2315 (9,9%)	1087 (5,8%)	2315 (19,5%)	1025 (10,6%)	80 (0,9%)	62 (0,7%)	699 (23,1%)	318 (8,7%)
Myocardial infarction	7.934 (1.9%)	2.529 (0.5%)	3.805 (1%)	1.303 (0.3%)	2.749 (15,6%)	827 (4,7%)	2739 (18%)	819 (5,7%)	10 (0,4%)	8 (0,2%)	1380 (5,9%)	399 (2,1%)	1380 (11,6%)	376 (3,9%)	37 (0,4%)	23 (0,3%)	362 (12%)	103 (2,8%)

Valvopathies	3.053 (0.7%)	3.815 (0.8%)	1.804 (0.5%)	2.540 (0.6%)	633 (3,6%)	707 (4%)	619 (4,1%)	670 (4,6%)	14 (0,6%)	37 (0,7%)	616 (2,6%)	568 (3%)	616 (5,2%)	473 (4,9%)	63 (0,7%)	95 (1,1%)	130 (4,3%)	117 (3,2%)
Peripheric artery disease	4.821 (1.2%)	2.077 (0.4%)	2.491 (0.7%)	1.399 (0.3%)	1.239 (7%)	399 (2,3%)	1227 (8,1%)	387 (2,7%)	12 (0,5%)	12 (0,2%)	1091 (4,7%)	279 (1,5%)	1091 (9,2%)	239 (2,5%)	78 (0,9%)	40 (0,5%)	250 (8,3%)	58 (1,6%)
Cancer history	23.997 (5.8%)	30.849 (6.5%)	18.091 (4.9%)	26.006 (6%)	2.401 (13,6%)	2.191 (12,4%)	2248 (14,8%)	1930 (13,3%)	153 (6,3%)	261 (5,1%)	3505 (15%)	2652 (14,2%)	3505 (29,5%)	1802 (18,6%)	993 (11,2%)	850 (9,6%)	576 (19%)	417 (11,4%)
Hereditary thrombophilia	186 (0%)	468 (0.1%)	131 (0%)	372 (0.1%)	39 (0,2%)	72 (0,4%)	34 (0,2%)	54 (0,4%)	5 (0,2%)	18 (0,4%)	16 (0,1%)	24 (0,1%)	16 (0,1%)	19 (0,2%)	1 (0%)	5 (0,1%)	5 (0,2%)	8 (0,2%)
Stroke	10.056 (2.4%)	10.326 (2.2%)	5.203 (1.4%)	5.759 (1.3%)	2.713 (15,4%)	2.902 (16,5%)	2696 (17,7%)	2859 (19,8%)	17 (0,7%)	43 (0,8%)	2140 (9,2%)	1665 (8,9%)	2140 (18%)	1564 (16,2%)	127 (1,4%)	101 (1,1%)	511 (16,9%)	416 (11,4%)
Ischemic stroke	9.381 (2.3%)	9.586 (2%)	4.734 (1.3%)	5.159 (1.2%)	2.644 (15%)	2.827 (16%)	2630 (17,3%)	2790 (19,3%)	14 (0,6%)	37 (0,7%)	2039 (8,7%)	1564 (8,4%)	2039 (17,2%)	1490 (15,4%)	96 (1,1%)	74 (0,8%)	499 (16,5%)	401 (11%)
Hemorrhagic stroke	1.025 (0.2%)	1.070 (0.2%)	708 (0.2%)	775 (0.2%)	137 (0,8%)	150 (0,9%)	134 (0,9%)	143 (1%)	3 (0,1%)	7 (0,1%)	180 (0,8%)	145 (0,8%)	180 (1,5%)	114 (1,2%)	38 (0,4%)	31 (0,4%)	32 (1,1%)	26 (0,7%)
Acute liver disease	60 (0%)	43 (0%)	52 (0%)	33 (0%)	3 (0%)	6 (0%)	2 (0%)	6 (0%)	1 (0%)	0 (0%)	5 (0%)	4 (0%)	5 (0%)	2 (0%)	2 (0%)	2 (0%)	0 (0%)	0 (0%)
CKD	8.894 (2.2%)	9.423 (2%)	5.467 (1.5%)	6.166 (1.4%)	1.359 (7,7%)	1.532 (8,7%)	1310 (8,6%)	1470 (10,2%)	49 (2%)	62 (1,2%)	2068 (8,9%)	1725 (9,2%)	2068 (17,4%)	1409 (14,5%)	360 (4,1%)	316 (3,6%)	394 (13%)	368 (10,1%)
COPD	12.500 (3%)	5.462 (1.2%)	8.026 (2.2%)	4.138 (1%)	1.634 (9,3%)	575 (3,3%)	1543 (10,1%)	521 (3,6%)	91 (3,8%)	54 (1,1%)	2840 (12,2%)	749 (4%)	2840 (23,9%)	554 (5,7%)	707 (8%)	195 (2,2%)	530 (17,5%)	146 (4%)
Asthma	33.175 (8.1%)	44.799 (9.5%)	30.711 (8.3%)	40.596 (9.4%)	1.005 (5,7%)	2.024 (11,5%)	825 (5,4%)	1515 (10,5%)	180 (7,4%)	509 (10%)	1459 (6,3%)	2179 (11,6%)	1459 (12,3%)	1301 (13,4%)	748 (8,5%)	878 (9,9%)	221 (7,3%)	374 (10,2%)
Multiple sclerosis	536 (0.1%)	1.208 (0.3%)	467 (0.1%)	1.098 (0.3%)	30 (0,2%)	40 (0,2%)	25 (0,2%)	26 (0,2%)	5 (0,2%)	14 (0,3%)	39 (0,2%)	70 (0,4%)	39 (0,3%)	31 (0,3%)	16 (0,2%)	39 (0,4%)	5 (0,2%)	8 (0,2%)
Parkinson's disease	1.926 (0.5%)	2.199 (0.5%)	1.157 (0.3%)	1.448 (0.3%)	304 (1,7%)	354 (2%)	287 (1,9%)	321 (2,2%)	17 (0,7%)	33 (0,6%)	465 (2%)	397 (2,1%)	465 (3,9%)	291 (3%)	102 (1,2%)	106 (1,2%)	77 (2,5%)	76 (2,1%)
Dementia	2.812 (0.7%)	7.786 (1.7%)	1.649 (0.4%)	5.157 (1.2%)	434 (2,5%)	1.436 (8,1%)	404 (2,7%)	1314 (9,1%)	30 (1,2%)	122 (2,4%)	729 (3,1%)	1193 (6,4%)	729 (6,1%)	918 (9,5%)	145 (1,6%)	275 (3,1%)	105 (3,5%)	225 (6,1%)
Alzheimer	2.625 (0.6%)	4.169 (0.9%)	776 (0.2%)	2.828 (0.7%)	185 (1%)	716 (4,1%)	172 (1,1%)	634 (4,4%)	13 (0,5%)	82 (1,6%)	323 (1,4%)	625 (3,3%)	323 (2,7%)	464 (4,8%)	75 (0,8%)	161 (1,8%)	43 (1,4%)	116 (3,2%)
IBD	2.824 (0.7%)	2.940 (0.6%)	2.504 (0.7%)	2.692 (0.6%)	132 (0,7%)	122 (0,7%)	113 (0,7%)	86 (0,6%)	19 (0,8%)	36 (0,7%)	188 (0,8%)	126 (0,7%)	188 (1,6%)	73 (0,8%)	89 (1%)	53 (0,6%)	28 (0,9%)	13 (0,4%)
